# Effects of Transgenic Cry1Ac + CpTI Cotton on Non-Target Mealybug Pest *Ferrisia virgata* and Its Predator *Cryptolaemus montrouzieri*

**DOI:** 10.1371/journal.pone.0095537

**Published:** 2014-04-21

**Authors:** Hongsheng Wu, Yuhong Zhang, Ping Liu, Jiaqin Xie, Yunyu He, Congshuang Deng, Patrick De Clercq, Hong Pang

**Affiliations:** 1 State Key Laboratory of Biocontrol, School of Life Sciences, Sun Yat-sen University, Guangzhou, China; 2 Department of Crop Protection, Faculty of Bioscience Engineering, Ghent University, Ghent, Belgium; French National Institute for Agricultural Research (INRA), France

## Abstract

Recently, several invasive mealybugs (Hemiptera: Pseudococcidae) have rapidly spread to Asia and have become a serious threat to the production of cotton including transgenic cotton. Thus far, studies have mainly focused on the effects of mealybugs on non-transgenic cotton, without fully considering their effects on transgenic cotton and trophic interactions. Therefore, investigating the potential effects of mealybugs on transgenic cotton and their key natural enemies is vitally important. A first study on the effects of transgenic cotton on a non-target mealybug, *Ferrisia virgata* (Cockerell) (Hemiptera: Pseudococcidae) was performed by comparing its development, survival and body weight on transgenic cotton leaves expressing Cry1Ac (Bt toxin) + CpTI (Cowpea Trypsin Inhibitor) with those on its near-isogenic non-transgenic line. Furthermore, the development, survival, body weight, fecundity, adult longevity and feeding preference of the mealybug predator *Cryptolaemus montrouzieri* Mulsant (Coleoptera: Coccinellidae) was assessed when fed *F. virgata* maintained on transgenic cotton. In order to investigate potential transfer of Cry1Ac and CpTI proteins via the food chain, protein levels in cotton leaves, mealybugs and ladybirds were quantified. Experimental results showed that *F. virgata* could infest this bivalent transgenic cotton. No significant differences were observed in the physiological parameters of the predator *C. montrouzieri* offered *F. virgata* reared on transgenic cotton or its near-isogenic line. Cry1Ac and CpTI proteins were detected in transgenic cotton leaves, but no detectable levels of both proteins were present in the mealybug or its predator when reared on transgenic cotton leaves. Our bioassays indicated that transgenic cotton poses a negligible risk to the predatory coccinellid *C. montrouzieri* via its prey, the mealybug *F. virgata*.

## Introduction

Genetically modified (GM) crops hold great promise for pest control [1]–[4]. Most popular GM crops express one or more toxin genes from bacteria such as *Bacillus thuringiensis* (Bt), trypsin inhibitors such as cowpea trypsin inhibitor (CpTI), plant lectins, ribosome-inactivating proteins, secondary plant metabolites, vegetative insecticidal proteins and small RNA viruses [5]–[7]. So far Bt-cotton has been commercialized in the United States (1996), Mexico (1996), Australia (1996), China (1997), Argentina (1998), South Africa (1998), Colombia (2002), India (2002), Brazil (2005), and Burkina Faso (2008) and occupies 49% of the total global cotton area [8], [9]. To delay the development of pesticide resistance in the major cotton pests [7], the bivalent transgenic cotton cultivar (CCRI41) expressing Cry1Ac and CpTI, has been commercially available since 2002 in China [10]. Currently, the cotton cultivar CCRI41 is planted at a large scale in the Yellow river cotton area in China [11]. However, with the rapid expansion in the commercial use of GM plants, there is an increasing need to understand their possible impact on non-target organisms [12]–[14]. Non-target effects of several cultivars (Cry1Ac + CpTI cotton) on beneficial arthropods including pollinator insects have been recently studied [11], [15]–[21].

Most studies on the potential ecological impacts of transgenic plants on phloem-feeding insects have focused on aphids or whiteflies [4], [22]–[27]. Studies on the interactions between mealybugs and GM crops have not been previously reported. Like aphids and whiteflies, mealybugs are obligate phloem feeders. Several species of mealybugs have caused considerable economic damage to agricultural and horticultural plants in the tropics in the last few decades [28]. They also have the potential to become major cotton pests which is evident from the severe damage reported in different parts of Asia [29]–[31]. Particularly, *Phenacoccus solenopsis* Tinsley (Hemiptera: Pseudococcidae) has attracted much attention worldwide because of its harmful effects on cotton [30], [32]–[35]. Indeed, this pest can successfully thrive on both Bt-cotton and non-Bt cultivars of cotton [36]. However, *P. solenopsis* is not the only mealybug species that infests cotton in Asia. Also *Maconellicoccus hirsutus* (Green) has increasingly been reported infesting cotton in India and Pakistan [37], [38]. Mealybugs are attacked by a range of specialist predators and parasitoids. These non-target species can thus be exposed to GM toxins by feeding on or parasitizing their prey or host [39]–[41] and there may be side effects on the behavior of these natural enemies [12], [42]. Therefore, there is a need to evaluate the potential effects of transgenic cotton on mealybugs and their key natural enemies.

The striped mealybug, *Ferrisia virgata* (Cockerell) (Hemiptera: Pseudococcidae), is also a cosmopolitan and polyphagous species that attacks a wide variety of crops including cotton [34], [43], [44]. The adult female is wingless, and has an elongated body covered by a powdery white wax, with a pair of dark longitudinal stripes on the dorsum and white wax threads extending from the posterior end resembling tails [34]. In cotton, *F. virgata* occurs in patches and feeds on all parts of a plant, particularly on growing tips or on leaves [33]. The species has been found infesting colored fiber cotton and has emerged as a serious pest in the Northeast of Brazil [34]. Given that mealybugs like *P. solenopsis*, *M. hirsutus* and *F. virgata* are aggressive invasive pests that seriously threaten cotton production, significant concern over their potential effects on transgenic cotton should be raised. At present, only the cotton mealybug *P. solenopsis* has been reported to damage Bt cotton. However, whether other mealybug species can infest transgenic cotton is yet to be determined.

The mealybug destroyer, *Cryptolaemus montrouzieri* Mulsant (Coleoptera: Coccinellidae), is a ladybird native to Australia and has been used in many biological control programs as one of the most efficient natural enemies to suppress mealybug outbreaks around the world [45]–[47]. Both the adults and larvae of the ladybird prey on a variety of mealybugs [47]. *C. montrouzieri* has also been used as a biological control agent in areas where outbreaks of *F. virgata* and *P. solenopsis* occur [38], [48]–[50]. These predators can encounter transgene products expressed by plants (Bt toxins) when feeding on plant material such as pollen, nectar, or leaf exudates and when preying on organisms that have consumed transgenic plant tissue or toxin-loaded prey [51]–[53].In the present study, bioassays were performed to assess the development, reproduction and feeding choices of *C. montrouzieri* presented with mealybugs reared on the cotton cultivar CCRI41 versus its near-isogenic non-transgenic line. To study whether Cry1Ac and CpTI proteins can pass through the trophic chain up to a natural enemy, quantification of Cry1Ac and CpTI proteins in leaves, mealybugs and ladybirds was also done.

This study is the first report on tritrophic relationships involving a non-target pest mealybug (*F. virgata*), its predator (*C. montrouzieri*) and a transgenic cotton cultivar expressing Cry1Ac (Bt toxin) and CpTI (Cowpea Trypsin Inhibitor).

## Materials and Methods

### Plants

Bivalent transgenic cotton cultivar CCRI 41 (Bt+CpTI cotton) and non-transgenic cotton cultivar CCRI 23 (control) were used as the host plants in all experiments. CCRI 41 was bred by introducing the synthetic Cry1Ac gene and modified CpTI (cowpea trypsin inhibitor) gene into the elite cotton cultivar CCRI 23 by way of the pollen tube pathway technique [54]. Seeds of transgenic Cry1Ac and CpTI cotton cultivar CCRI 41 and its near-isogenic CCRI 23 were obtained from the Institute of Cotton Research, Chinese Academy of Agricultural Sciences. Both cultivars were planted singly in plastic pots (16×13 cm) with the same soil. All plants were individually grown from seeds in climate chambers (25±1°C, 75±5% RH, 16: 8 h (L: D)) and they were five weeks old (about five to eight true leaves) at the start of experiments.

### Insects

Stock cultures of *C. montrouzieri* and *F. virgata* were originally obtained from the State Key Laboratory of Biocontrol, Sun Yat-sen University, Guangzhou, China. Cultures of *C. montrouzieri* were reared on *Planococcus citri* Risso (Hemiptera: Pseudococcidae) and *F. virgata*, which were both produced on pumpkin fruits (*Cucurbita moschata* (Duch.ex Lam.) Duch. ex Poiretand) in metal frame cages (45×36×33 cm) covered with fine-mesh nylon gauze. The colony of *F. virgata* was maintained on plastic trays (40×30 cm) containing pumpkins as food. Environmental conditions at the insectarium were 26±2°C, 50±10% RH and a photoperiod of 16: 8 h (L: D). Both *C. montrouzieri* and *F. virgata* cultures used in these experiments had been maintained at our facilities for at least six years.

### Bioassay with *F. Virgata*

#### Effects of transgenic Cry1Ac and CpTI cotton on development and survival of *F. virgate*

Development and survival of *F. virgata* on the leaves of transgenic and non-transgenic cotton plants was studied in climate chambers (25±1°C, 75±5% RH, 16: 8 h (L: D)). The experiment was subdivided into two stages: crawlers (first instars) of the mealybug were reared for the first 5 days in 6-cm diameter plastic containers to preclude escape, whereas in a second stage larger plastic bags were used to accommodate the later instars. In the first stage of the experiment 20 newly emerged first-instar nymphs (<24 h) springing from the same female were placed in a plastic container (6.0×1.5 cm) covered with a fine-mesh nylon gauze using a soft paintbrush. Each plastic container had a small hole in it allowing a leaf to be inserted. A piece of cotton wool was wrapped around the petiole to prevent *F. virgata* from escaping through the hole in the container. To encourage crawlers to settle, the environmental chamber was maintained in complete darkness for 24 h [55], [56]. All plastic containers were fixed on live cotton plants by small brackets. Mealybugs on each cotton plant represented a cohort or a replicate. A total of 15 cohorts (replicates) were prepared for both the treatments with transgenic and control cotton plants.

In the second stage of the experiment the mealybugs were kept in transparent plastic bags (15×10 cm) with several small holes for ventilation. The transparent plastic bag together with cotton wool wrapped around the petiole could also prevent mealybugs from escaping or dropping off. The mealybug cohorts on each leaf (still attached to the plant) were examined every 12 h, and the development and survival of each nymphal instar were recorded. Successful development from one instar to the next was determined by the presence of exuviae. Survival rate of each stage was calculated as the percentage of individuals that successfully developed to the next stage in a cohort [56]. The sex of individual mealybugs could not be determined at the crawler stage. Therefore, sex was determined during the latter part of the second instar when males change their color from yellow to dark. At this point, the developmental times of males and females were recorded separately [55].

#### Effects of transgenic Cry1Ac and CpTI cotton on body weight of *F. virgate*

To assess the body weights of *F. virgata*, 200 second-instar nymphs were collected at the same time from stock cultures reared on pumpkin. Ten mealybugs per cotton plant were placed as a cohort on the leaves of 10 non-transgenic or transgenic cotton plants using a soft paintbrush. Thus, a total of 10 cohorts (replicates) were prepared for both the treatments with transgenic and control cotton plants. To prevent mealybugs from escaping or dropping off, each leaf infested with *F. virgata* was placed in a transparent plastic bag (15×10 cm) with several small holes for ventilation. To encourage the nymphs to settle, the environmental chamber was maintained in complete darkness for 24 h. Thereafter, the plants were kept in an environmental chamber as described above. Surviving mealybugs from the initial 10 individuals on each plant were weighed individually after 10 and 20 days using an electronic balance (Sartorius BSA124S, Germany) with a precision of 0.1 mg.

### Tritrophic Bioassay with *C. Montrouzieri*

#### Effects of transgenic Cry1Ac and CpTI cotton on the development and survival of immature *C.montrouzieri*

Two plastic boxes (12.0×5.0×4.0 cm, covered with fine-mesh nylon gauze for ventilation) each containing 50 *C. montrouzieri* eggs (<12 h old) collected from the stock colony were placed in a climate chamber (25±1°C, 75±5% RH, 14∶10 h (L:D) photoperiod). The eggs were observed carefully every 12 h and numbers of larvae that hatched were recorded. Newly hatched first-instar larvae from 50 *C. montrouzieri* eggs (<12 h old) were individually transferred to the leaves of non-transgenic (45 larvae) or transgenic cotton (46 larvae), which were previously infested with *F. virgata* (∼60–100 mealybugs per leaf). Each cotton plant received two or three *C. montrouzieri* larvae which were distributed on different leaves. Pieces of cotton wool were wrapped around the stem or petiole to prevent the larvae from leaving the cotton leaves. Predator larvae were randomly moved to newly infested plants when mealybug prey was depleted. In total, about 60 non-transgenic or transgenic cotton plants were used for the experiment. Larvae of *C. montrouzieri* were checked every 12 h for molting, which was determined by the presence of exuviae. The developmental time and survival of each immature stage of *C. montrouzieri* were also recorded up to adulthood.

#### Effects of transgenic Cry1Ac and CpTI cotton on reproduction and adult longevity

After adult emergence, *C. montrouzieri* females and males were single paired and each pair was transferred to a transparent plastic bag (15×10 cm) with several small holes for ventilation. A total of 12 and 16 pairs (replicates) were set up for non-transgenic and transgenic cotton plants, respectively. A piece of cotton was placed in the bag for oviposition. A leaf of non-transgenic or transgenic cotton infected with *F. virgata* (∼60–100 mealybugs per leaf) was also placed in this bag. The bag containing *C. montrouzieri* adults was transferred to a new freshly infested leaf on the same plant every 3 days. The pre-oviposition period, number of eggs and survival of the mating pairs of *C. montrouzieri* were checked every day until the death of all adults.

#### Effects of transgenic Cry1Ac and CpTI cotton on body weight of *C. montrouzieri*

In order to determine fresh body weight during each developmental stage, 50 newly hatched first instar *C. montrouzieri* (<12 h old) were individually transferred to the leaves of non-transgenic or transgenic cotton using a soft hairbrush and placed in close vicinity to the prey. The leaf with mealybugs (∼60–100 mealybugs per leaf) was replaced every 3 days and *C. montrouzieri* larvae were checked every 12 h for molting and development. Newly emerged 1^st^, 2^nd^, 3^rd^, and 4^th^ instar larvae, pupae and adults of *C. montrouzieri* were weighed individually after 24 h using an electronic balance (Sartorius BSA124S, Germany) with a precision of 0.1 mg to record their body mass.

#### Feeding performance of *C. montrouzieri* on mealybugs reared on non-transgenic versus transgenic cotton leaves

Metal frame cages (45×36×33 cm) covered with fine-mesh nylon gauze were used in these experiments with five cages or replicates each. In each cage, 20 *C. montrouzieri* adults (10 males and 10 females, <1 month old) were taken from the laboratory stock and starved for 24 h. Three pots each of non-transgenic and transgenic cotton (with one cotton plant per pot) were placed in a cage. Each non-transgenic or transgenic cotton plant was previously infested with 20 similar-sized female adult mealybugs. Every day, the plants infected with 20 mealybugs were replaced with newly infested plants. The experiment continued for 9 days and the numbers of consumed mealybugs were recorded every day.

### Quantification of Toxins in Leaves, *F. Virgata* and *C. Montrouzieri*

To confirm Cry1Ac and CpTI expression of the transgenic cotton plants (8-leaf stage) used in both bioassays, five leaf samples were collected from five different cotton plants. Each sample was obtained from a middle-upper leaf of a transgenic or control plant [57]. Approximately 100 mg fresh weight (f.w.) of the transgenic or control cotton leaves was collected.

To quantify the level of Cry1Ac and CpTI in *F. virgata,* a group of approximately sixty gravid females from the laboratory culture were allowed to settle on cotton leaves and reproduce. After 24 h, about 100 newborn nymphs were brushed carefully onto each transgenic or control cotton leaf and the leaf was covered with a transparent plastic bag (15×10 cm) with several small holes for ventilation. A piece of cotton wool was wrapped around the petiole to prevent *F. virgata* from escaping from the leaf. To encourage crawlers to settle, the environmental chamber was maintained in complete darkness for 24 h. Three weeks later, five samples of *F. virgata* larvae (with a total fresh weight of 60–100 mg) were collected from plants of either variety.

To assess the potential transfer of Cry1Ac and CpTI proteins via the food chain, a transgenic or control cotton leaf (still attached to the plant) which was previously infested with *F. virgata* as described above and a newly molted 2^nd^ instar larva or an adult (<1 month old) of *C. montrouzieri* were kept in a ventilated plastic bag (15×10 cm). Ten transgenic or control cotton plants were used. After 3 days, five samples of individual *C. montrouzieri* larvae or adults were collected for analysis.

All experiments described above were conducted in a growth chamber at 25±1°C, 75±5% RH and a photoperiod of 16∶8 h (L∶D). All samples were weighed and transferred to 1.5-ml centrifuge tubes. Samples were kept at −20°C until quantification of Cry1Ac and CpTI proteins.

The amount of Cry1Ac protein in the leaf and insect material was measured using an enzyme linked immuno-sorbent assay (ELISA). Envirologix Qualiplate Kits (EnviroLogix Quantiplate Kit, Portland, ME, USA) were used to estimate Cry1Ac quantities. The quantitative detection limit of the Cry1Ac kit was 0.1 ng ml^−1^. The ELISA polyclonal kits used to detect CpTI protein were obtained from the Center for Crop Chemical Control, China Agricultural University (Beijing, China). The method has been validated [58] and the limit of detection and working range of the assay were 0.21 and 1–100 ng ml^−1^, respectively [59]. Prior to analysis, all insects were washed in phosphate buffered saline with Tween-20 (PBST) buffer to remove any Cry1Ac and CpTI toxin from their outer surface. After adding PBST to the samples at a ratio of about 1∶10 (mg sample: µl buffer) in 1.5 ml centrifuge tubes, the samples were fully ground by hand using a plastic pestle. To detect Cry1Ac protein, samples were centrifuged for 5 min at 13,000×g and leaf samples were diluted to 1∶10 with PBST (insect samples were not diluted). For analysis of CpTI protein, the tubes were centrifuged at 10,000×g for 15 min. The supernatants were used to detect targeted proteins. ELISA was performed based on the manufacturer’s instructions. ODs were calibrated by a range of concentrations of Cry1Ac or CpTI made from purified toxin solution.

### Data Analysis

For the studied parameters in the bioassay with *F. virgata*, the average values of each cohort were used as replicates for the data analyses.The duration of the immature stages, survival and weight on transgenic and non-transgenic cotton were compared using independent t-tests. For the tritrophic bioassay with *C. montrouzieri*, a Mann–Whitney U test was performed for the duration of the immature stages and preoviposition period. Weights, fecundity, oviposition period, and adult longevity were analyzed using independent t-tests. The percentages of total survival and egg hatch were compared by logistic regression, which is a generalized linear model using a probit (log odds) link and a binomial error function [60]. Each test consists of a regression coefficient that is calculated and tested for being significantly different from zero, for which P-values are presented [61]. Consumption rates in the feeding performance test were compared using a general linear model for repeated measures analysis of variance (ANOVA) followed by a LSD test. All datasets were first tested for normality and homogeneity of variances using a Kolmogorov-Smirnov test and Levene test, respectively, and transformed if necessary. SPSS software (IBM SPSS Statistics, Ver. 20) was used for all statistical analyses. For all tests, the significance level was set at P≤0.05.

## Results

### Bioassay with *F. Virgata*

#### Effects of transgenic Cry1Ac and CpTI cotton on the developmental duration of *F. virgate*

*F. virgata* nymphs completed their development when reared on non-transgenic cotton CCRI 23 and its near-isogenic transgenic cotton CCRI 41 (Table 1). However, there was no significant difference in the developmental duration of female or male *F. virgata* larvae reared on transgenic or non-transgenic cotton except during the first and fourth instars. The duration of first instar development was longer on transgenic cotton. In contrast, fourth instar males reared on transgenic cotton had shorter development compared to those reared on non-transgenic cotton. No significant differences were observed in the developmental durations of the second instar, third instar and in cumulative developmental time.

**Table 1:** Mean number of days (±SE) for each developmental stage of *F. virgata* reared non-transgenic or transgenic cotton leaves.

Developmental time per stage (days)							Cumulative (days)	
Cotton cultivar	First†	Second		Third		Fourth*	Female	Male
		Female	Male	Female	Male	Male		
Non-transgenic cotton	8.24±0.16a	5.31±0.19a	6.34±0.28a	6.43±0.07a	2.48±0.09a	5.68±0.08a	19.98±0.23a	22.55±0.24a
Transgenic cotton	8.72±0.14b	5.10±0.16a	6.62±0.23a	6.59±0.14a	2.39±0.07a	5.26±0.14b	20.41±0.36a	22.87±0.22a
t	−2.218	0.846	−0.776	−0.982	−0.063	2.654	−1.007	−1.008
df	28	28	28	28	28	28	28	28
P	0.035	0.405	0.444	0.334	0.405	0.013	0.323	0.322

Means ± SE within a column followed by the same letter are not significantly different (P>0.05; independent t-test). The experiment was started with 15 cohorts (replicates) per treatment.

† Sex could not be determined before the second instar.

*Female mealybugs have only three nymphal instars while males have four nymphal instars.

#### Effects of transgenic Cry1Ac and CpTI cotton on nymphal survival of *F. virgate*

No significant difference was observed in the survival rate of female or male *F. virgata* nymphs reared on transgenic or non-transgenic cotton except in the first instar (Table 2). The survival rate of the first instars was lower when reared on transgenic cotton. No significant differences in the survival rates of the second instar, third instar, fourth instar of male and in cumulative survival rate were observed.

**Table 2:** Mean (±SE) survival rate (%) of each developmental stage of *F. virgata* reared on non-transgenic or transgenic cotton leaves.

Cotton cultivar	First†	Second	Third		Fourth*	Total survival
			Female	Male	Male	
Non-transgenic cotton	75.67±3.68a	83.56±3.71a	94.93±3.06a	87.98±4.53a	97.38±1.86a	57.00±4.05a
Transgenic cotton	61.33±4.15b	86.96±4.20a	89.79±2.93a	97.22±1.94a	97.00±2.06a	49.00±4.37a
t	2.583	−0.592	1.213	1.878	0.006	1.343
df	28	28	28	28	28	28
P	0.015	0.501	0.235	0.071	0.892	0.190

Means ± SE within a column followed by the same letter are not significantly different (P>0.05; independent t-test). The experiment was started with 15 cohorts (replicates) per treatment.

† Sex could not be determined before the second instar.

*Female mealybugs have only three nymphal instars while males have four nymphal instars.

#### Effects of transgenic Cry1Ac and CpTI cotton on body weight of *F. virgate*

The weight of all *F. virgata* nymphs increased when reared on transgenic or non-transgenic cotton leaves for 10 or 20 days. However, nymphal weights were not significantly influenced by cotton variety (P>0.05, independent t-tests). Mean weights (± SE) of adult *F. virgata* reared on non-transgenic cotton (77 and 52, respectively) and transgenic cotton (87 and 48, repectively) leaves for 10 days were 1.29±0.15 mg and 1.30±0.10 mg (t = −0.091; df = 18; P = 0.928), and for 20 days were 2.30±0.22 mg and 2.06±0.21 mg (t = 0.799; df = 18; P = 0.434), respectively.

### Tritrophic Bioassay with *C. Montrouzieri*

#### Effects of transgenic Cry1Ac and CpTI cotton on development and survival of immature *C. montrouzieri*

The developmental time of all immature stages and total survival did not differ when reared on transgenic or its near-isogenic non-transgenic cotton (Table 3). There was no significant difference in immature stages and survival.

**Table 3:** Developmental time (days) and total survival rate (%) of the immature stages of *C. montrouzieri* reared non-transgenic or transgenic cotton leaves.

Cotton cultivar	Developmental time per stage (days)*						Total survival (%)^†^
	1^st^ instar	2^nd^ instar	3^rd^ instar	4^th^ instar	Pupa	Total immature	
Non-transgenic cotton	3.12±0.04	2.74±0.05	3.22±0.06	5.50±0.07	8.71±0.07	23.40±0.13	80.00±0.06
Transgenic cotton	3.11±0.03	2.81±0.05	3.27±0.04	5.49±0.07	8.66±0.09	23.31±0.09	86.96±0.05
U/χ^2^	942.5	776.0	820.0	878.0	640.5	672.5	0.798
df	1	1	1	1	1	1	1
P	0.793	0.293	0.538	0.973	0.610	0.610	0.372

No significant difference was observed between the control and treated groups within the same column (means ± SE) (P>0.05; *Mann-Whitney U test or **^†^**Wald χ^2^ test); 45and 46 larvae were initially tested for non-transgenic and transgenic cotton plants, respectively.

#### Effects of transgenic Cry1Ac and CpTI cotton on body weight of *C. montrouzieri*

**Figure 1:**
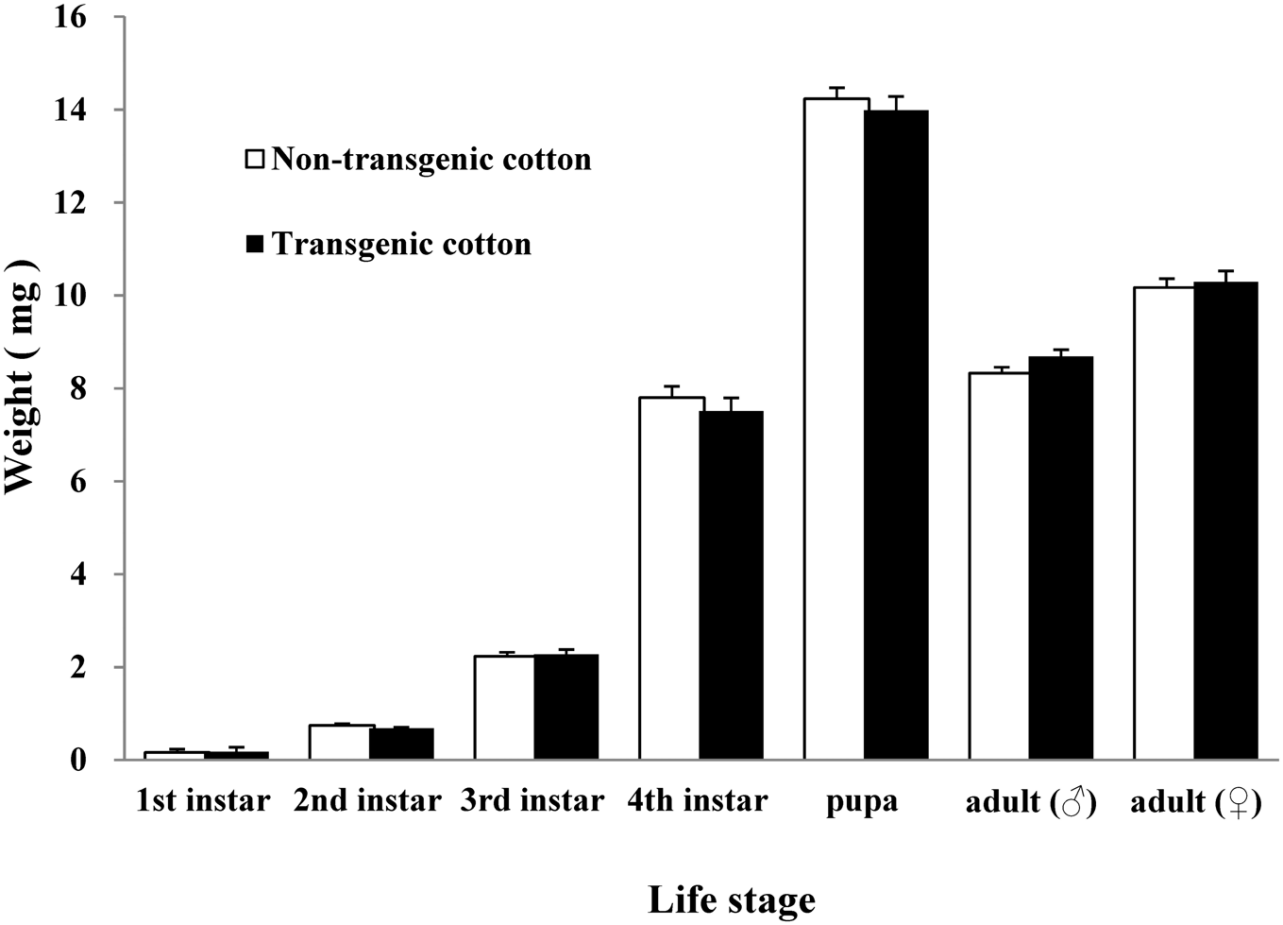
Weight upon molting (means ± SE) of different life stages of *C. montrouzieri* reared on non-transgenic or transgenic cotton leaves. No significant difference was observed between the control and treated groups in each life stage (P>0.05; independent t-test). The experiment was started with 50 larvae per treatment.

When reared on transgenic cotton, first instar (t = −1.579; df = 8; P = 0.153), second instar (t = 1.941; df = 98; P = 0.055), third instar (t = −0.343; df = 97; P = 0.733) and fourth instar larvae (t = 0.782; df = 95; P = 0.436), pupae (t = 0.659; df = 90; P = 0.512), and male (t = −1.795; df = 39; P = 0.080) and female (t = −0.421; df = 34; P = 0.677) adults showed no significant difference in their body weight upon emergence compared with their counterparts reared on non-transgenic cotton (Figure 1).

#### Reproduction and longevity of *C. montrouzieri* reared on non-transgenic or transgenic cotton leaves

Preoviposition period (U = 68; df = 1; P = 0.906), fecundity (t = 0.390; df = 21; P = 0.700), number of eggs laid per female per day (t = 1.581; df = 21; P = 0.129), egg hatch (χ^2^ = 1.753; df = 1; P = 0.185), male longevity (t = 0.148; df = 26; P = 0.883) and female longevity (t = −1.183; df = 26; P = 0.247) were not significantly affected by treatment (Table 4).

**Table 4:** Reproduction and longevity of *C. montrouzieri* females reared on non-transgenic or transgenic cotton leaves.

Cotton cultivar	Preoviposition period (days)^‡^	Fecundity (eggs/♀)*	Oviposition rate (eggs/♀/day)*	Egg hatch (%)^†^	Longevity (days)*	
					♂	♀
Non-transgenic cotton	7.00±0.93	823.80±84.25	7.21±0.83	90.60±0.01	160.96±17.36	131.42±9.82
Transgenic cotton	7.18±0.58	766.46±110.99	5.44±0.74	90.80±0.11	157.88±11.62	152.91±13.85

No significant difference was observed between the control and treated groups within the same column (Means ± SE) (P>0.05; *independent t-test, **^‡^**Mann-Whitney U test or **^†^**Wald χ^2^ test); 12 and 16 pairs of *C. montrouzieri* were used for non-transgenic and transgenic cotton plants, respectively.

#### Feeding performance of *C. montrouzieri* on mealybugs reared on non-transgenic versus transgenic cotton leaves

**Figure 2:**
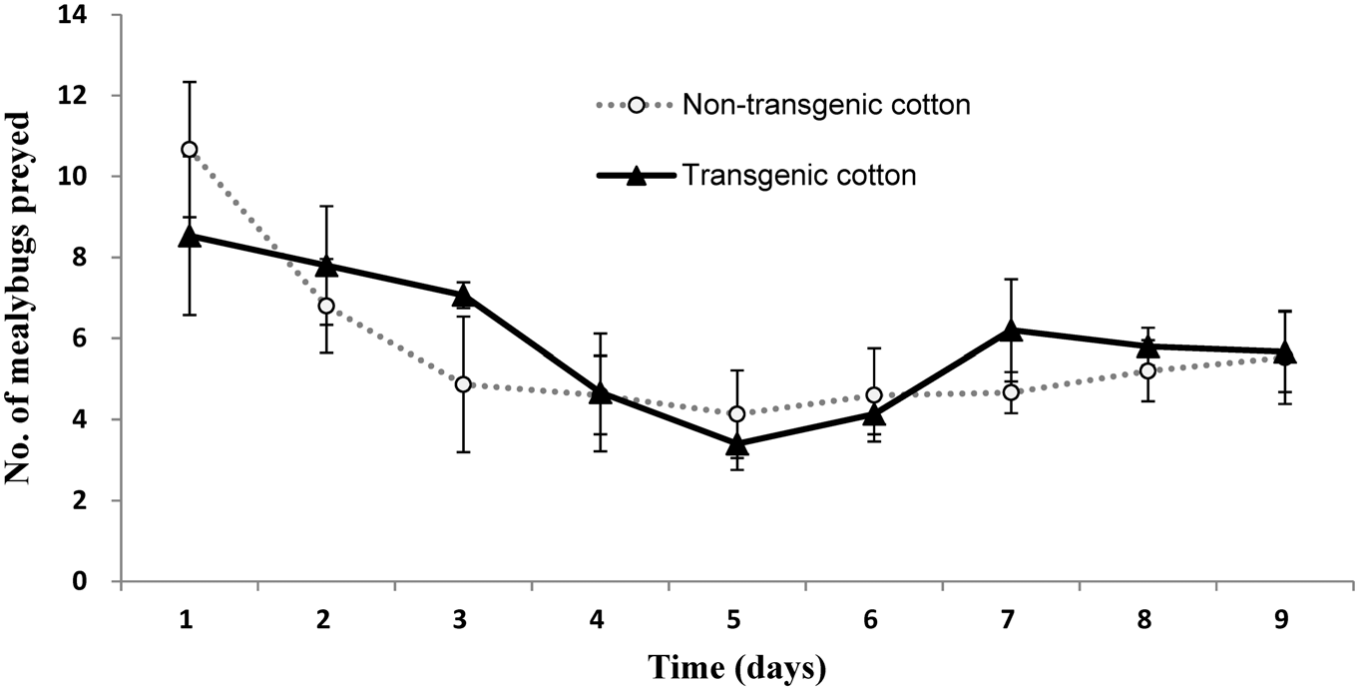
Feeding performance of *C. montrouzieri* on *F. virgata* mealybugs reared on non-transgenic or transgenic cotton. The data represent numbers (±SE) of mealybugs consumed per individual predator over a 9 day period (P>0.05; repeated measures analysis of variance (ANOVA) followed by a LSD test).

Daily consumption of mealybugs by *C. montrouzieri* adults on non-transgenic cotton was not different from that on transgenic cotton during the entire 9-day test period (F = 0.111; df = 1; P = 0. 748) (Figure 2). The interaction between the factors cotton type and time was also not significant, meaning that differential consumption of mealybugs between transgenic cotton and non-transgenic cotton was not a function of time (F = 0.692; df = 8; P = 0.697). However, *C. montrouzieri* consumed a decreasing number of mealybugs on both cotton varieties over the course of the experiment (F = 5.098; df = 8; P<0.001).

### Quantification of Toxins in Leaves, *F. Virgata* and *C. Montrouzieri*

Expressed levels of the Cry1Ac and CpTI proteins in CCRI41 cotton leaves averaged 5.76±0.33 µg Cry1Ac/g f.w. and 14.28±1.70 ng CpTI/g f.w. (means **±** SE), respectively. ELISA revealed that *F. virgata* maintained on transgenic cotton did not contain detectable amounts of the Cry1Ac and CpTI proteins. Similarly, no Cry1Ac or CpTI protein was detected in *C. montrouzieri* larvae and adults. None of the non-transgenic cotton leaves, or of the mealybug and ladybird samples reared on control plants were found to contain any Cry1Ac or CpTI protein.

## Discussion

*F. virgata* is a widely spread mealybug and is reported in more than 100 countries around the world, including the USA, Argentina, Canada, India, China, Brazil, and Pakistan [44], where transgenic cotton is being cultivated. Our results demonstrate that *F. virgata* nymphs completed their development when reared on leaves of both non-transgenic and transgenic cotton. Overall, no significant differences were detected in the total survival, cumulative developmental duration and body weight of the immature stages of *F. virgata* reared on transgenic and non-transgenic cotton. Higher mortality was observed during the first instar on transgenic cotton but the difference was small and total mortality from first instar to adult did not differ between treatments. These results indicate that the transgenic Bt+CpTI cotton had negligible adverse effects on the development of *F. virgata*, which is consistent with previous reports by Dutt [36] and Zhao et al. [18] stating that the mealybug *P. solenopsis* was able to infest Bt and Bt+CpTI transgenic cotton without negative effects on its fitness.

Further, ELISA analyses revealed that none of the mealybug samples from the Bt+CpTI cotton contained detectable Bt protein despite high expression levels in leaves. Like aphids and whiteflies, mealybugs are obligate phloem sap feeding insects. We postulate that *F. virgata* was not exposed to the Bt endotoxins expressed in the cotton plants given its phloem feeding habit. In previous studies on transgenic maize, Bt toxins were not detected or only in negligible amounts in the phloem sap, or in aphids that had fed on the maize [62], [63]. In transgenic Bt cotton fields the density of sap-feeding insects, such as whiteflies, aphids and leafhoppers, has been reported to be higher than in non-transgenic cotton fields [64], [65]. Lawo et al. [26] noted that Indian Bt cotton varieties had no effect on aphids, leading them to conclude that Bt cotton poses a negligible risk for aphid antagonists and that the aphids should remain under natural control in Bt cotton fields.

On the other hand, it was expected that any impact of transgenic Bt+CpTI cotton on mealybugs may be largely attributed to the CpTI gene encoding the cowpea trypsin inhibitor, which acts on insect gut digestive enzymes and inhibits protease activity [66]. The cysteine protease inhibitor, oryzacystatin I (OC-I), was detected in both leaves and phloem sap of transgenic oilseed rape, which significantly inhibited growth of *Aphis gossypii* Glover, *Acyrthosiphon pisum* (Harris), and *Myzus persicae* (Sulzer) in vitro, despite low levels of proteolysis in the guts of these homopterans [67]. Although in the present study no CpTI protein could be detected by ELISA in *F. virgata* samples, the effects of the CpTI protein on the mealybug cannot be fully excluded. Low amounts of the cowpea trypsin inhibitors (CpTI) ingested by *F. virgata*, could act as an anti-feedant to the mealybugs, which may explain lower survival rates in the first instar. In fact, Han et al. [11] demonstrated an antifeedant effect of CCRI41 cotton pollen (Bt+CpTI) on the honey bee *Apis mellifera* L. Feeding behaviour of the bees was disturbed and they consumed significantly less CCRI41 cotton pollen than in the control group given conventional cotton pollen. The antifeedant affect may have led to insufficient food uptake and malnutrition for the larvae and newly emerged bees [11], [68], [69]. Further, according to an EPG (Electric Penetration Graph) signal, Liu et al.[23] found that the frequencies of moving and searching for feeding sites, and probing activity of the aphid *A. gossypii* reared on CCRI 41cotton were significantly higher than those on control cotton. Given their high mobility 1^st^ instar mealybugs are responsible for plant colonization in the field [70]. When 1^st^ instars of *F. virgata* select their feeding site on transgenic cotton a succession of walks and stops is observed. Consequently, the 1^st^ instar mealybugs in our study may have spent more energy in finding and probing for food on transgenic cotton leaves than on non-transgenic leaves, which might have negatively affected the survival rates in the first instar. However, if present, this antifeedant effect to *F. virgata* appears limited because no significant difference was found in total survival and developmental duration. Besides, the *F. virgata* clones used in the present study were not resistant to the transgenic plants, as they had been maintained exclusively on pumpkin for at least 6 years without any contact with cotton. Due to inadvertent adaptations to laboratory conditions, host finding and acceptance behaviors of mass produced insects may be changed over the generations [71]–[73]. Colonization effects may therefore have influenced the responses of the mealybug to cotton as a host plant and it may be warranted to investigate the interactions between transgenic cotton and wild or recently colonized mealybugs.

The mealybug destroyer, *C. montrouzieri,* might ingest toxins expressed by transgenic plants that accumulate in the mealybugs feeding on these plants. In this context, we conducted tritrophic bioassays to investigate the potential effects of CCRI 41 cotton on *C. montrouzieri* by using *F. virgata* as prey. These experiments did not reveal any adverse effects on the fitness of *C. montrouzieri* after ingestion of *F. virgata* that fed on Bt+CpTI cotton leaves compared with those that fed on the corresponding non-transgenic cotton leaves. Besides a longer oviposition period on transgenic cotton than on non-transgenic cotton, there were no differences in reproductive parameters. This finding is consistent with other studies which reported no or little adverse effects on various predators or parasitoids after feeding on different Bt + CpTI cottons, including a ladybird [74] and two hymenopteran parasitoids [15], [75].

Several possible mechanisms can explain the observed results. Firstly, *C. montrouzieri* may not be sensitive to Cry1Ac proteins. Porcar et al. [76] reported no statistical differences in mortality of *C. montrouzieri* adults and *Adalia bipunctata* L. larvae fed on artificial diets with or without Cry1Ab and Cry3Aa toxins. Duan et al. [77] and Lundgren and Wiedenmann [78] found no significant adverse effects when Bt maize pollen were fed to larvae of the ladybird *Coleomegilla maculata* DeGeer. The same ladybird species was also found to be unaffected by Bt cotton or higher amounts of Cry2Ab and Cry1Ac proteins indicating that Bt cotton poses a negligible risk to *C. maculata* [57]. In addition, no negative effects of Bt-transgenic plants were observed on the development, survival, and reproduction of the ladybirds *Hippodamia convergens* (Guérin-Méneville) and *Propylea japonica* (Thunberg) through their aphid prey that fed on the Bt plants [25], [79].

In the field, no significant differences were observed in the abundance of coccinellid beetles on Bt-transgenic and non-transgenic cottons [80]. Pollen from Cry1Ac+CpTI transgenic cotton (CCRI41) did not affect the pollinating beetle *Haptoncus luteolus* (Erichson) in the field and in the laboratory [19]. Xu et al. [81] found that CCRI41 cotton did not affect the population dynamics of non-target pests and predators including ladybirds and spiders in Xinjiang, China. Zhang et al. [82] observed negative effects on the ladybird *P. japonica* when offered young *Spodoptera litura* (F.) larvae reared on Bt-transgenic cotton expressing Cry1Ac toxin; however, adverse effects on the ladybird were attributed to poor prey quality. Lumbierres et al. [83] investigated the effects of Bt maize on aphid parasitism and the aphid–parasitoid complex in field conditions on three transgenic varieties and found that Bt maize did not alter the aphid–parasitoid associations and had no effect on aphid parasitism and hyperparasitism rates.

Ramirez-Romero et al. [84] concluded that Bt-maize did not affect the development of the non-target aphid *Sitobion avenae* (F.) and Cry1Ab toxin quantities detected in these aphids were nil, indicating that none or negligible amounts of Cry1Ac are passed on from the aphids to higher trophic levels. Probably, the amount of Cry1Ac/CpTI proteins ingested by the mealybugs in our study was too low to be effective. Indeed, ELISA measurements indicated that Bt+CpTI cotton-reared *F. virgata* and its predator did not contain detectable amounts of the Cry1Ac and CpTI protein. Because the commercial ELISA kit for determining CpTI expression was not available [19] or the amount of CpTI proteins was lower than the lowest limit of quantification [11], [21] there are few earlier reports on tritrophic interactions involving CpTI protein. On the contrary, many studies related to the transfer of Bt toxic proteins to higher trophic levels have been carried out. For example, ELISA analyses revealed no or only trace amounts of Bt protein in sap-sucking insects of the order Hemiptera after feeding on different Bt plants, including maize [63], [84]–[86] and cotton [26], [87]. Trace amounts of Bt toxins were detected in *A. gossypii* feeding on Bt cotton cultivars and ladybirds preying on Bt-fed aphids [41]. Another possible reason for the weak effect of Cry1Ac/CpTI proteins is that ladybirds may digest or excrete the toxins taken up via their prey. For example, Li and Romeis [88] fed the ladybird *Stethorus punctillum* (Weise) with spider mites, *Tetranychus urticae* (Koch), reared on Cry3Bb1-expressing Bt maize. Subsequent bioassays revealed that the Cry protein concentrations in the ladybird beetle larvae and adults were 6- and 20-fold lower, respectively, than the levels in the spider mite prey. Cry1 proteins were also detected in *C. maculata* when offered *Trichoplusia ni* (Hübner) larvae reared on Bt-cotton, but the Bt protein levels were 21-fold lower for Cry2Ab and 6-fold lower for Cry1Ac compared to the concentrations in the prey [57].

In summary, our study indicates that *F. virgata* can successfully develop on bivalent transgenic cotton CCRI41expressing Cry1Ac+CpTI and thus can pose a risk for this crop. The finding that not only *P. solenopsis* but also other mealybugs like *F. virgata* can easily infest transgenic cotton plants has important implications for pest management in this cropping system. Further, our study demonstrates that transgenic cotton poses a negligible risk to the predatory coccinellid *C. montrouzieri* via its mealybug prey. However, further field studies assessing the impact of transgenic cotton on the mealybug pest and its key natural enemies are needed.
